# Recommendations for Intersectoral Collaboration for the Prevention and Control of Vector-Borne Diseases: Results From a Modified Delphi Process

**DOI:** 10.1093/infdis/jiaa404

**Published:** 2020-10-29

**Authors:** Carl Abelardo T Antonio, Amiel Nazer C Bermudez, Kim L Cochon, Ma Sophia Graciela L Reyes, Chelseah Denise H Torres, Sophia Anne S P Liao, Dorothy Jean N Ortega, Abegail Visia Marie C Silang, Deinzel R Uezono, Evalyn A Roxas, Maria Sonia S Salamat

**Affiliations:** 1 Department of Health Policy and Administration, College of Public Health, University of the Philippines, Manila, Philippines; 2 Department of Applied Social Sciences, Hong Kong Polytechnic University, Kowloon, Hong Kong; 3 Department of Epidemiology and Biostatistics, College of Public Health, University of the Philippines, Manila, Philippines; 4 Department of Epidemiology, School of Public Health, Brown University, Providence, Rhode Island, USA; 5 Department of Statistics, Chinese University of Hong Kong, Sha Tin, Hong Kong; 6 College of Public Health, University of the Philippines Manila, Manila, Philippines; 7 Duke-NUS Graduate Medical School, Singapore; 8 Research Institute for Tropical Medicine, Muntinlupa City, Philippines; 9 Department of Medical Microbiology, College of Public Health, University of the Philippines, Manila, Philippines; 10 Department of Medicine, College of Medicine and Philippine General Hospital, University of the Philippines, Manila, Philippines

**Keywords:** intersectoral collaboration, vector-borne diseases, consensus, Delphi technique

## Abstract

**Background:**

Intersectoral collaboration in the context of the prevention and control of vector-borne diseases has been broadly described in both the literature and the current global strategy by the World Health Organization. Our aim was to develop a framework that will distill the currently known multiple models of collaboration.

**Methods:**

Qualitative content analysis and logic modeling of data abstracted from 69 studies included in a scoping review done by the authors were used to develop 9 recommendation statements that summarized the composition and attributes of multisectoral approaches, which were then subjected to a modified Delphi process with 6 experts in the fields of health policy and infectious diseases.

**Results:**

Consensus for all statements was achieved during the first round. The recommendation statements were on (1–3) sectoral engagement to supplement government efforts and augment public financing; (4) development of interventions for most systems levels; (5–6) investment in human resource, including training; (7–8) intersectoral action to implement strategies and ensure sustainability of initiatives; and (9) research to support prevention and control efforts.

**Conclusions:**

The core of intersectoral action to prevent vector-borne diseases is collaboration among multiple stakeholders to develop, implement, and evaluate initiatives at multiple levels of intervention.

Vector-borne diseases claim a significant toll on human health and social development. In 2017, approximately 650 000 deaths were attributable to the 3 major mosquito-borne diseases alone (malaria, dengue, and yellow fever), a figure that represents about 7.7% and 1% of the estimated mortality from communicable diseases and mortality from all causes in the same year, respectively [1]. In addition to its effect on human health, these conditions have also been shown to result into economic harm at the household and societal levels [2–5].

A cornerstone in the control of vector-borne diseases is the Integrated Vector Management strategy, a decision-making process developed and advanced under the principles of cost-effectiveness, intersectoral action, regulatory and operational measures, subsidiarity, decision-making, and sustainability [6–8]. The lack of intersectoral collaboration has been identified as a key reason for the failure to significantly curb the burden of vector-borne diseases, as intersectorality was identified as a mechanism to foster sustainability of the Integrated Vector Management approach [9–11]. This insight, among others, informed the development of the World Health Organization’s “Global Vector Control Response 2017–2030” that calls for strengthening “inter- and intra-sectoral action and collaboration” to help achieve the goal of reducing mortality from vector-borne diseases by 75% by 2030 [12].

Intersectoral collaboration within the context of vector-borne diseases has been broadly described in both the literature and the current global strategy, making translation into action at the regional, country, and even subnational levels challenging. Furthermore, the effectiveness of these collaborative arrangements has also not been consistently measured, nor were the contributions of different participating sectors assessed [13]. A scoping review undertaken by the authors in 2017 for the Special Programme for Research and Training in Tropical Diseases (TDR), as part of a landscape analysis to better understand intersectoral collaboration, showed that there were multiple models of collaboration described in the literature and that there was a need to develop a general framework to better inform the development of multisectoral approaches for the prevention and control of vector-borne diseases [14]. We build on the results of this scoping review by reporting on the development and validation of 9 key recommendations for intersectoral collaboration.

## METHODS

Data for this study were abstracted from results of a scoping review [15–17] that we published previously [14]. In brief, the review attempted to address the research question, “What models of intersectoral collaborations have been implemented in countries where vector-borne diseases are a priority issue, and what are documented effects on the prevention of vector-borne diseases?” Inclusion criteria were the following: any type of record (eg, case studies, reviews, commentaries) with an accessible full-text copy in the English language, published between 1 January 1985 and 31 December 2016, and which discusses the population, concept, and context parameters contained in the research question. No hand searching was done, and articles that required purchase to access their full-text versions were not included. The scoping review yielded a total of 7535 records, 69 of which were included in the synthesis. The authors recognize the limitation in the selection of references due to publication language restriction, which was implemented due to feasibility constraints. Nonetheless, it is worth pointing out that despite use of a broad search strategy across 4 databases, as well as inclusion of gray literature, only about 1% of the total record yield was deemed relevant to the scoping review question, and the included papers discussed intersectoral collaboration across a broad range of countries in North America, South America, Africa, and Asia. Thus, the exclusion of non-English-language references may not have a substantial impact on the generalizability of the paper’s findings.

Qualitative content analysis was carried out using NVivo 11 Pro (Version 11.3; QSR International Pty Ltd, 2016) to identify the following components of intersectoral collaboration as documented in the literature: goals and outcomes, strategies and activities, resource requirements, and policy and implementation gaps. A modified logic model [18–20] was then used to consolidate data on the types of resources, stakeholders, and sources of funds that are needed to roll out strategies that cover advocacy, health education, and capacity building, among others. Expected outputs and outcomes of intersectoral collaborations were derived from those of successfully implemented and consistently monitored strategies described in the literature. We also identified antecedent and mediating factors that negatively affect the outcome of the collaborative arrangements. Given the purpose of analysis, a logic model was deemed an appropriate tool to organize the different data points extracted from the included literature as it is able to reflect a theory of change, or a description of how interventions are supposed to convert inputs, or resources and actions, to expected outcomes, or results.

The results were converted into 9 recommendation statements that summarized the composition and attributes of effective multisectoral approaches. These statements, with corresponding evidence notes, were then subjected to a modified Delphi process [21], with 2 rounds as the limit and with consensus defined a priori by a supermajority for each items (ie, 70% of the participants either agree or strongly agree with the findings). Statements were emailed to the Delphi participants using Google Forms for rating using a 4-point Likert scale (1 = strongly disagree to 4 = strongly agree). Six experts, all of whom are medical doctors in the fields of health policy and infectious diseases, were identified by the senior review team members and invited to participate in the Delphi consensus process. Two panelists came from Indonesia and are experts in the fields of health policy and community health, 1 panelist is from Thailand and is an expert on infectious diseases (dengue in particular), and the other 3 panelists reside in the Philippines, all infectious and tropical disease specialists.

## RESULTS

This section summarizes the results of the Delphi process. Because consensus for all statements was achieved during the first round (ie, 100% agreement among Delphi participants), a second round was no longer conducted. Each recommendation statement is followed by a parenthetical note on the level of agreement achieved from the Delphi process. This is followed by a synthesis of the evidence supporting the recommendation statement.

** Recommendation statement 1:** Engagement of various industries and civil society organizations is needed to supplement the efforts of ministries of health and multilateral organizations for the prevention and control of vector-borne diseases (*Level of agreement:* strongly agree = 100%).

A lack of adequate and continuous financial support as well as logistical barriers to the accessibility of drugs and other vector-targeted and immunological interventions were identified as major gaps in administration of prevention and control programs for vector-borne diseases. Transportation was one of the key needs highlighted due to a scarcity in vehicles for logistical purposes and a need for access to remote populations. These problems are magnified by the decline in financial support from foreign aid and local government, and weakening of political support from multilateral organizations (ie, United Nations Children’s Fund, Roll Back Malaria). This calls for other sources of support, which can be given by industries and civil society organizations.

An outcome of collaboration strategies identified in the articles included in the synthesis was that as the number of communities and other stakeholders engaged in the control programs increased, implementation became easier. Sustainability also showed marked improvement, and activities were expanded to other areas.

**Recommendation statement 2:** Mobilization of support from private industries and other nonhealth sectors (ie, education, agriculture, businesses) is needed to augment existing public financing of initiatives for the prevention and control of vector-borne diseases (*Level of agreement*: strongly agree = 50%, agree = 50%).

There is a need for mobilization of resources and support from various sectors to enhance the financing of vector-borne disease prevention activities. Out of the 30 funding sources for prevention and control programs identified in our scoping review, 17 were from the public sector, 8 from the private sector, and 5 from mixed sources (both public and private). The respective governments of the different countries were the most frequent source, having funded 12 programs.

**Recommendation statement 3:** Innovative financing mechanisms need to be developed and negotiated between donors and recipients to ensure that this fits the local context (*Level of agreement:* strongly agree = 50%, agree = 50%).

Despite the financial support given by the public and private sectors, insufficient budget is still one of the most common problems encountered by the programs. Self-sufficiency can be achieved by the programs through revenue-generating projects. Some programs have developed alternative income-generating schemes (eg, fish production) that have successfully augmented their budget.

**Recommendation statement 4:** Required interventions for the prevention and control of vector-borne diseases are those that influence most interpersonal, organizational, community, and policy aspects of the system (*Level of agreement:* strongly agree = 50%, agree = 50%).

Strategies related to health education and promotion, adequate training, and public health at the interpersonal, organizational, community, and policy levels showed satisfactory outcomes and impact. These outcomes include but are not limited to increased awareness about malaria and dengue, increased research initiatives for their prevention and control, and decreased incidence and prevalence rates.

**Recommendation statement 5:** Development of human resources across all sectors is a critical investment to ensure that efforts aimed at the health, environment, economic and educational aspects of prevention and control programs for vector-borne diseases can be sustained over time (*Level of agreement:* strongly agree = 100%).

Human resources were found to be the most frequent type of resources used by intersectoral collaborations. These include health workers, entomology and environmental experts, world leaders, government officials, teachers, students, parents, researchers, technicians and operators, and community members and volunteers, among others. However, human resource management deficiencies were collectively shown to be a major gap in the sustainability of prevention and control programs for vector-borne diseases. These deficiencies include migration of professionals due to better employment opportunities outside the country, a difficulty in the recruitment of doctors to affected areas, and a loss in continuity in partnerships due to high turnover of staff. Inadequate staff training and local capacity were also shown to be a major hindering factor leading to the failure of malaria control strategies.

**Recommendation statement 6:** Innovative training programs that target local health professionals and community members are necessary to build a workforce that will ensure the sustainability of prevention and control programs for vector-borne diseases (*Level of agreement:* strongly agree = 67%, agree = 33%).

Training programs are shown to be effective on all 4 levels of the social ecological model of health promotion; methods include direct guidance of household members by collaborators to practice simple mosquito control methods, the recruitment and support of graduate students for research projects, teacher training for school-based interventions, and community and field training for local health professionals and community volunteers.

Little to no training has been given for personnel in the periphery such as community health workers, district medical officers, and municipal health teams regarding the analysis of epidemiological data and communication strategies. A shortage of skilled and knowledgeable personnel in control planning and management is a major roadblock to the success of vector-borne disease control interventions.

**Recommendation statement 7:** Intersectoral action is a necessary component in the implementation of core and support strategies to prevention and control efforts—that is, social mobilization, integrated vector management, capacity building, communication strategies, policy development, resource mobilization, and other public health measures (*Level of agreement:* strongly agree = 100%).

Based on the outcomes of the programs, collaboration among different sectors has led to easier implementation and management of the strategies employed. Participation in the various activities of the programs increased with the help of the government and the community, among others.

**Recommendation statement 8:** Sustainability of initiatives for the prevention and control of vector-borne diseases emanate from shared activities and mutual agreements between and among stakeholders, to include local governments, national government agencies, multilateral organizations, academe, business, industry, agriculture, and the community at large (*Level of agreement:* strongly agree = 100%).

Bilateral agreements that divide responsibilities between the local government and a foreign aid agency were noted to have resulted to an unsustainable program. Support given by the government and by the community addressed this gap, showing improved sustainability of programs. Collaborations among the stakeholders also led to the expansion of the programs to other geographic areas.

**Recommendation statement 9:** Comprehensive health research spanning epidemiological and entomological disciplines, among others, is necessary for the development of specific vector-borne disease control measures, policies, and programs (*Level of agreement:* strongly agree = 83%, agree = 17%).

Effective health research strategies were shown to include the innovation and development of new drugs, insecticides, models of disease epidemiology, vaccines, and mosquito traps, as well as research on malaria epidemiology and the entomology of its vector.

There is currently a lack of data and information on the epidemiology of malaria as well as incompleteness of surveillance coverage, which impedes the planning and evaluation of vector-borne disease control programs. This is accompanied by a lack of research capacity, specifically poor understanding of malaria epidemiology and biology and an insufficiency in knowledge on the implementation of effective control measures on the community level.

## DISCUSSION

This article set out to describe a framework for intersectoral collaboration in the context of vector-borne diseases prevention and control efforts, developed from results of a scoping review and expert consensus. Intersectoral collaboration (ie, concerted effort from ministries of health, multilateral organizations, business and industry, civil society, and the community at large) at various administrative and operational levels has been identified as a crucial component to support implementation of interventions and ensure sustainability of prevention and control efforts for vector-borne diseases.

The recommendations provided in this paper are embedded in 2 important models currently influencing public health discourse, both of which trace their roots to systems theory. First, intersectoral action is about the synergy and convergence of efforts across multiple dimensions (ie, within the health sector, across sectors that influence the upstream determinants of health, transcending administrative boundaries), an idea developed in the “Health in All Policies” framework [22–24], and identified as a key approach to attaining the Sustainable Development Goals [25, 26]. The focus of action for intersectoral action, on the other hand, should not be confined to addressing needs of individuals affected by vector-borne diseases, but should also be directed toward institutional and structural factors [27, 28]. In short, intersectoral action can be defined as bringing together multiple actors and stakeholders to develop, implement, and evaluate initiatives at multiple levels of intervention.

We view these recommendations as having wide applicability since the statements have been derived from a synthesis of experience and empirical evidence on the implementation of intersectoral approaches from different countries and validated through a consensus approach. The consensus process with an external group was deemed necessary as a means of validating the recommendation statements that were formulated based on the author team’s own understanding and interpretation of the empirical evidence derived from the literature. Furthermore, while the recommendation statements can be construed as “generic” in nature, it is worth noting, for example, that most intersectoral initiatives included in our prior scoping review [14] had a preponderance of policy- and community- level strategies, hence the fourth recommendation on the need to expand strategies to influence the other system levels. Likewise, strategies related to the prevention and control of vector-borne diseases are human resource intensive; however, the quantity and quality of staff was a recurring issue among the collaborative initiatives identified, which prompted the formulation of the fifth and sixth recommendation on human resource development. We recognize, however, that there is a limited number, and sectoral and geographic representation, in our pool of experts. Nonetheless, the recommendation statements may still have obtained the same consensus with a broader group given their general nature.

Translation into actionable strategies, tactics, and activities needs to be embedded within the local context. The extent to which these recommendations will apply must be done in consultation with community and government leaders. For example, the statement on the investment in human resource development cannot be transformed into policy without identifying the number of existing local staff, as well as the baseline skill sets and knowledge of health professionals and community members. Financial innovations must also fit with the capacity of community leaders, volunteers, and local industries to ensure sustainability. The prerequisites of communication among all stakeholders and the development of plans from both ends of the spectrum (bottom-up and top-down) in spite of and because of existing political environments are applicable for all recommendations for multisectoral approaches, and will incorporate trust into the foundation for inclusive and sustainable interventions.

From a research perspective, there is a need for testing of the applicability of the recommendation statements into the real-world conditions and further refine, elaborate, and validate their cogency, as well as elucidate the underlying mechanism that will lead to translation of the recommendations into important outcomes in vector-borne disease prevention and control. Furthermore, given the limitation of the consensus process employed in this article, primary collection of data using interviews or focus groups may be considered for future research to identify other possible barriers and enablers of multisectoral action.

## Notes

***Acknowledgments*.** Dr Qingxia Zhong and Dr Florence Fouque (both of the UNICEF/UNDP/World Bank/WHO Special Programme for Research and Training in Tropical Diseases) provided comments and suggestions on an earlier version of this manuscript.

***Financial support.*** This commissioned review was supported by TDR, and the International Development Research Centre (IDRC), www.idrc.ca (contract number 301713).

***Supplement sponsorship.*** This Supplement is sponsored by TDR: the Special Programme for Research and Training in Tropical Diseases co-sponsored by the United Nations Children’s Fund (UNICEF), the United Nations Development Programme (UNDP), the World Bank and the World Health Organization (WHO).

***Potential conflicts of interest.*** C. A. T. A. reports professional fees from Johnson & Johnson (Philippines), outside the submitted work. All other authors report no conflicts of interest.
